# Different and Unequal: A Qualitative Evaluation of Salient Factors Influencing Energy Intake in Adults with Overweight and Obesity

**DOI:** 10.3390/nu11061365

**Published:** 2019-06-18

**Authors:** Maria Carlota Dao, Ellen Messer, Teresa Conigliaro, Kylie Sakaida, Alexis F. Ouellette, Victoria Himaras, Sophie Thiron, Susan B. Roberts

**Affiliations:** 1 Jean Mayer USDA Human Nutrition Research Center on Aging, Tufts University, Boston, MA 02111, USA; ksakaida@bu.edu (K.S.); alouellette@gmavt.net (A.F.O.); himarasv@merrimack.edu (V.H.); Susan.Roberts@tufts.edu (S.B.R.); 2 Friedman School of Nutrition Science and Policy, Tufts University, Boston, MA 02111, USA; Ellen.Messer@tufts.edu (E.M.); Teresa.Conigliaro@tufts.edu (T.C.); 3 Institut de Recherche pour le Développement, 34060 Montpellier, France; sophie.thiron@wanadoo.fr

**Keywords:** eating behavior, weight management, weight status, externality

## Abstract

Environmental factors such as food availability and variety can function as cues for overeating in individuals susceptible to overweight or obesity, but relatively little is known about other types of environmental factors that may also be important. This qualitative study compared and contrasted categories of internal and external cues through focus groups and key informant interviews with 24 adults (26 to 77 years old) in the United States who had a body mass index within the healthy range (21.6 ± 2.5 kg/m^2^) or had overweight or obesity (29.1 ± 3.6 kg/m^2^). Five domains of external factors influencing food intake were identified: (a) Environmental cues including food availability and variety; (b) normative expectations for dietary intake; (c) food palatability; (d) overt social pressures to overeat; and (e) perceived social expectations around eating. All external domains were noted by participants with overweight or obesity to be challenging, and solutions to avoid overeating were lacking; however, overt social pressures and perceived social expectations appeared to be especially problematic. By explicitly defining different domains of external factors that challenge healthy weight regulation, this study identifies specific targets to address in interventions for healthy weight management.

## 1. Introduction

The prevalence of obesity continues to increase worldwide, and overeating is recognized to be an important factor in both weight gain and the maintenance of excess weight [1]. Both physiological factors such as hunger and satiety [2,3], and external factors arising from the modern “toxic” food environment may contribute to overeating, but external factors are widely believed to play a dominant role in the increasing prevalence of obesity over time [4,5,6,7]. This is because food attributes such as high energy density [8], wide variety [9,10,11], large portion size [12,13,14,15], food processing to enhance food reward and palatability [16], and liquid versus solid state foods [17] typically promote overeating, and these factors have become more prevalent worldwide in parallel with rising rates of obesity [6]. 

However, not all individuals living in a toxic food environment become obese, and some that do are able to lose weight without weight regain, raising the question of how internal and external contributors to energy intake differ between individuals who maintain excess weight and those who do not. Herman and Polivy proposed a boundary model of human eating behavior in which hunger and satiety do influence food intake but only at the extremes of eating, while environmental factors are more typical daily cues for food consumption [18]. Differences in these boundaries between individuals could underlie different susceptibilities to obesity. This suggestion is consistent with the earlier externality theory of obesity proposed by Schachter [19], which postulates that individuals with a susceptibility to obesity are particularly sensitive to environmental, or ‘external’, cues, with resulting overeating, weight gain, and maintenance of the obese state. However, beyond food-related cues such as portion size and food availability [4,5,9,10,12,13], there has been little research to identify different categories of factors contributing to externality-mediated overeating. 

The purpose of this study was to obtain information through key informant interviews and focus groups with adults with a healthy body mass index (BMI) or who had overweight or obesity, with the goal of comparing and contrasting categories of externality factors that are perceived to contribute to overeating in individuals in different BMI categories. 

## 2. Materials and Methods

### 2.1. Study Population

Participants were a convenience sample recruited at the Jean Mayer USDA Human Nutrition Research Center on Aging (HNRCA) at Tufts University through the Metabolic Research Unit’s volunteer database for a cross-sectional qualitative study consisting of key informant interviews and focus groups on various topics around behaviors, attitudes, and customs relating to food, eating, and weight management. Inclusion criteria were older than 18 years, English proficiency and literacy, and availability to participate in one of the qualitative study-related activities. Prospective participants were provided with an electronic link to a screening questionnaire, in which they entered information about their age, gender, height, weight, race, time availability to schedule an interview or focus group, and contact information. BMI category was considered for recruitment. Participants with a healthy, overweight, or obese BMI were recruited, with emphasis on overweight/obese BMI to ensure that a wide variety of opinions and attitudes would be captured in this BMI category. For the key informant interviews, participants either had a healthy BMI (19–25 kg/m^2^, *n* = 5) or a BMI in the overweight or obese range (>25 kg/m^2^, *n* = 5). For the focus groups, there were 10 participants with an overweight BMI, and 4 participants with an obese BMI. Height and weight were based on self-reported values because precise information was not necessary for this study. There was one interview participant with a BMI close to 25 kg/m^2^, who was included in the overweight category. In the focus groups, there were seven participants with a BMI close to 25 kg/m^2^ included in the overweight category, and one participant with a BMI close to 30 kg/m^2^ included in the obese category. The protocol was determined to have exempt status after being reviewed and approved by the Tufts Health Sciences Institutional Review Board. All participants received an information form with details of the study, stating that participation was strictly voluntary, and a small honorarium.

### 2.2. Key Informant Interviews and Focus Groups

Ten semi-structured key informant interviews lasting approximately one hour were conducted at the HNRCA between August and September 2018 by the same nutrition scientist (MCD). In addition, three focus groups of 3 to 7 participants per group (*n* = 14 total participants), which lasted approximately 1.5 hours, were held between September and October 2018 and were moderated by the same researcher who also had a nutrition background (TC). All meetings were audio recorded and transcribed verbatim [20]. No identifying information was included in the transcriptions. 

**Figure 1:**
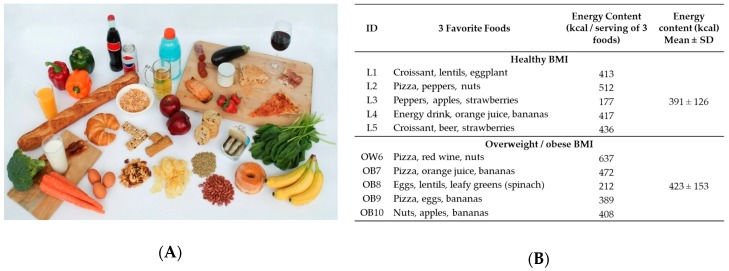
Ice breaker interview exercise. (**A**) Key informant interview participants were asked to identify their three favorite foods from this photograph. Included foods were selected to illustrate a broad range of foods from healthy to unhealthy based on their positive or negative association with risk of obesity and co-morbidities. (**B**) Favorite foods selected by participants, and individual and mean energy content per selection. SD, standard deviation. Source for calorie content: https://fdc.nal.usda.gov/.

Questions posed during the interviews and focus groups were designed by experts in nutrition, weight management, sociology, and anthropology. The questions were organized within seven domains for attitudes and behaviors relating to eating and weight management after a thorough literature review: (1) Significance of food (as sustenance, identity; covered during an ice-breaker exercise); (2) reasons for eating; (3) attitudes toward body image, nutrition, and health; (4) insularity, fatalism, and other general cultural traits; (5) meal patterns; (6) attitudes to hunger and restraint; and (7) external pressures to overeat/not eat. The specific questions that provided the starting point for the semi-structured discussions are shown in Table S1. In addition, key informant interviews began by asking subjects to select three foods they found most enjoyable from a photograph created for the study (ice-breaker, Figure 1). The foods were selected based on their positive or negative association with risk of obesity and cardiometabolic disease when included in habitual dietary intake [21,22,23,24,25,26,27,28,29,30]. 

### 2.3. Data Transcription and Analysis

Recordings were transcribed by three research assistants who also contributed to the coding. Transcribers and coders were blinded from the participants’ BMI status initially, while concepts, codes, and themes were being established [31,32]. The study leader performed quality control checks in randomly selected subsets of all the transcribed recordings. The transcripts were thoroughly read by all researchers involved in the analysis. Analysis began by sorting transcript data into the seven pre-existing domains described above, and coding key ideas that emerged from multiple close readings of these texts [33]. Codes were compiled into a code book along with definitions and examples for each code [31]. Emerging concepts and themes were discussed and finalized by the team. The coders reviewed and confirmed each other’s work through each step of the analysis. The study coordinator verified the work and harmonized the codes. Upon matching subjects’ transcripts with BMI, the data were categorized accordingly and content was compared across BMI categories. Microsoft Excel, Microsoft Word, and the software NVivo (NVivo qualitative data analysis software; QSR International Pty Ltd. Version 12, 2018) [34] were used for code book development, coding, and analysis. Mean and standard deviation (SD) are reported in quantitative tables.

## 3. Results

### 3.1. Population Characteristics

Participant characteristics are summarized in Table 1. Mean ± SD BMI for participants with a healthy weight was 21.6 ± 2.5 kg/m^2^, while it was 29.1 ± 3.6 kg/m^2^ for participants with overweight/obesity. Both groups had comparable age ranges, and most participants were >40 years (20 out of 24). There were 4 women in the healthy BMI group and 12 in the overweight/obese group. Individual age ranges and BMI categories for all participants can be found in Table S2.

When asked about their perception of their weight status, participants from different BMI categories gave diverging answers. Participants with a healthy BMI typically were not worried about their weight, as stated by participant L4: *“I haven’t been worried about my weight because I’m always active.”* In addition, participant L5 (formerly overweight) talked about a changed attitude after losing weight: *“I never used to [pay attention to weight] because I was much heavier and I just didn’t wanna know. But like over the past five or six months I, I pretty much tried to work out on a daily basis or as much as I could like consistently. And so I’ve lost a lot of weight and so I had it checked recently, um so I do now. I mean cause I’m not like “Oh no, I like weigh that much.”* In contrast, individuals with overweight or obesity did feel their weight had an impact on their health but frequently felt powerless to address it. For example, a participant in FG3OWOB said: *“I feel like if you gave me a questionnaire I would probably say my health is excellent but in fact I have lousy knees and I know if I lost twenty pounds I would have a better time with my knees and I think I eat quite healthily but probably too much of that healthy stuff…but anyway I’m not losing twenty pounds it seems.”* And participant OB7 expressed a similar view: *“Yeah I’m finding it difficult to lose weight I have to settle for maintaining weight that’s the best I can do at the moment.”* As did participant OB9: *“I have a weight loss goal but I didn’t put a time frame to it… I want to lose 20 lbs. But I didn’t put a time frame to it. I don’t want to be a stickler to that… I’m not gonna be hard on myself so that’s why I’m not…, jumping on the scale every day, like I used to be because I’m not gonna be so hard on myself if I see a pound up or a pound down, or if it’s not working quick enough. So, I just said ok I’m just gonna try to eat healthy, give myself a couple of months then step on the scale.”*

### 3.2. Food Preference: Link Between Pleasure and Healthfulness

Foods selected during the visual ice-breaker exercise given to participants in the key informant interviews are shown in Figure 1. Participants were asked to list their three “favorite foods” out of those shown on the food photograph (Figure 1A). The caloric content tended to be higher for the group with overweight/obesity than the healthy BMI group (Figure 1B), although statistical testing was not applied due to the small sample sizes. In several cases, particularly in the overweight/obese group, there was tension when selecting preferred foods based on palatability versus foods perceived to be the correct or healthy choice. For example, participant OW6 said: *“Well there are things I enjoy like pizza I am not eating because I don’t think it is as healthy as I would like it to be.”*

This statement contrasts one made by participant L3 in the healthy BMI group, describing how they prioritize healthy foods while also preparing/consuming foods they enjoy: L3: *“I’d say [health] that’s probably primary, that is, within the range of healthy food, then taste, and um for both of us but that is keeping within the range of healthy food… I rarely cook food that I don’t like.”* For both participants, health is an important criterion for food selection. However, L3 cooks and eats foods that they consider both healthy and palatable, while OW6 is aware that their food preferences, such as pizza, are not the healthiest choices.

In addition, concerns about weight management were often mentioned in this section of the interview, and some participants clearly connected this concern to their perceived difference between enjoyable foods (or foods they would have selected), and foods consumed more frequently in current attempts to manage their weight. This is illustrated by the following example from participant OB9: *“I would’ve circled the donut but I’m trying to lose my belly so I’m refraining from eating donuts.”* This participant’s favorite food choices were pizza, eggs, and bananas (Figure 1).

Overall, the prevalence of words related to restrictive behaviors around food was 56% higher in the interview transcripts from the overweight/obese group than the healthy BMI group, and this is illustrated in the following description by participant OB7: *“Well there’s virtually nothing I don’t like for reasons of taste. I have a very broad range of appetite. For reasons of health I **avoid** drinks with added sugar, I **avoid** sodas, I **avoid** meat for the most part, particularly processed meat. I **avoid** processed food, food with additives. Salt, I really don’t add salt to anything I eat and I try to **avoid** salt pretty much across the board as much as I can.”* These results represent the tension experienced between pleasure from eating and the perception of ideal eating habits, leading to attempts of behavior change through restriction of palatable foods in the group with overweight or obesity who, as described below, also experienced higher levels of disinhibition than the healthy BMI group.

In the focus groups, the ice-breaker exercise differed in that participants were asked to discuss and select three foods that they considered to be healthy and three foods they considered to be unhealthy. There was a general awareness in both interviews and focus groups about healthy dietary habits and foods considered part of a healthy diet, but few subjects expressed preference to consume these foods based on both palatability and nutritional value. For example, a focus group participant (FG1OW) said: *“They’re [selected foods] high sugar, high carbs, all the good stuff.”* and *“Ohhh. You can add that to the unhealthy group. That tastes good.”* These are additional examples of the tension generally found across participants with a higher BMI between pleasurable foods and foods they believe should be consumed for the improvement or maintenance of their health.

Thematic analysis of questions subsequent to the icebreaker led to the identification of behaviors around eating and weight management that were categorized into internal and external cues, and these are discussed separately below. 

### 3.3. Internal Cues and Eating Behavior Responses

Based on the interview and focus group responses as well as past research, five categories of internal cues were delineated: (1) Hunger and satiety, (2) restraint, (3) disinhibition, (4) cravings, and (5) association between pleasure and food composition. Participants talked about their reaction to hunger, satiety, and cravings in different situations, and how important these internal cues are relative to external factors. Hunger and satiety were the areas in which the two groups differed the most. As illustrated by typical participant quotes in Table 2, the group with overweight or obesity talked about the difficulties in managing hunger, which was framed on several occasions as a “problem,” as stated by participant OB7: *“I don’t have much patience and I do feel hungry frequently and that is probably a problem…sometimes think of hunger as being the root of my inability to lose weight and I occasionally glance at the ads for products that are being developed that allege that they address hunger. If I could safely take something or even eat in a certain way that would just make me less hungry during the course of the day I would jump at it…I see hunger as being kind of at the root of the problem, the inability to lose weight.”*

In contrast, the group with a healthy BMI did not describe hunger as a problem, and instead described their routine strategies to manage it, including consuming smaller meals and consistent eating schedules, and they used sensations of fullness as a signal to stop eating. They also mentioned feeling excessively full as a negative factor that limited intake. An example of this is provided by participant L1: *“So I don’t ever really get that hungry cause I’m always kind of, like I’ll take food with me. So I try to avoid getting really hungry because then I know I’ll probably, I might overeat.”* Therefore, there is an awareness of the challenge posed by an obesogenic environment by both groups, but different reactions to it. 

All participants described behaviors they use to restrain overeating, including focusing on healthy food and trying to reduce exposure to unhealthy food, for example, by keeping tempting foods out of the house. However, disinhibition was high in the group with overweight or obesity, who described eating in response to food availability: *“(OW6) At times I certainly eat too much… you make whatever amount … there is and you just eat it. I think that is very common. And maybe I have an idea in my mind what that should be. So it’s maybe more of my brain than in my stomach.”* This statement contrasts with one made by participant L1, who describes manipulating their food environment to eliminate unhealthy choices: *“(L1) I don’t buy them all the time, it is a favorite but I just don’t buy it all the time.”*

Responses to cravings also differed, with the healthy-weight participants appearing to embrace their pleasurable foods, but to avoid frequent access to these foods and enjoy them only occasionally: “(*L2) The thing that I really enjoy the most and I don’t buy it because I will eat the whole bag are Cheese Curls. That is my indulgence. I love Cheese Curls!… If I buy them I will only buy a small package because as I said I will eat the whole thing.”* In contrast, participants with overweight or obesity acted more on their cravings and did not describe restricting access to foods that they crave: *“(OB8) Uhhh. Yea, if it’s something I really enjoy, I don’t have to be hungry to eat it. I would just eat it. Not out of need to eat it … like my cottage cheese… So I might go in the fridgerator and grab the tub of cottage cheese and I might sit down there and eat it.”*

### 3.4. External Factors in Overeating

Participants discussed external factors potentially influencing overeating, including celebratory meals, feelings about eating alone versus eating with others or in public, and overt and perceived social expectations to overeat or undereat. Based on the responses, five categories of external cues were delineated: (1) The availability and variety of different foods in the environment, (2) normative cues for portion size in the environment and via dietary recommendations, (3) sensory food cues, (4) overt social pressures to overeat or undereat, and (5) perceived social expectations that caused overeating or undereating.

Representative quotes by participants are shown in Table 3. Descriptions of the challenges of living in an obesogenic environment were present across participants independent of BMI grouping, including food being more abundant during celebrations, and the importance of food palatability. However, participants with overweight or obesity consistently reported it was difficult to avoid overeating when faced with these external factors, such as large portions, food variety, and food offerings (e.g., hors d’oeuvres), as stated by participant OW6: *“I’ll eat it now if it’s available. You know, if I walked through some reception party, yes, always.”*

In addition, in response to overt social pressures to overeat, participants in this group specifically noted that there were situations where it would be insulting to not eat the food and that they would ‘force’ themselves to eat. For example, participant OB9 said: *“It depends on the situation. Like if I’m somewhere and I know I’m gonna be insulting them if I don’t eat it. I’d swallow without chewing. That’s about it and I’d never try again. Like I tried salad before and never again. Like little things like that. Roast beef at a friend’s house and I didn’t want to be insulting and say “oh I don’t eat that.” So I just swallowed it and didn’t even chew it.”* Conversely, participants in the healthy BMI group strategized about how to not respond to overt social pressures to overeat by disguising not eating, as portrayed by participant L3: *“But at a celebration I feel like I ought to have a drink, again I will go ask for seltzer with lime because everybody assumes you’re drinking a gin and tonic.”* However, as in the participants with a higher BMI, they also appeared vulnerable to overeating in response to perceived social expectations, which seemed pervasive, for example having a dessert at celebrations: *“(L5) “Um, but depends on the setting like, if I’m out to eat with my friends and they’re all eating dessert, then I’m more likely to get something like that. Um, I guess it depends on the setting, if it’s in a restaurant or at home. Um, like when I’m on my own I’ll just basically eat mostly healthy.”*

Participants with overweight and obesity most commonly expressed a feeling of comfort and not feeling judged when eating alone, which was linked to unrestricted behaviors, as stated by OB7: *“I just think when one is alone, you know you can sort of dive in be you know, be ravenous not you know perhaps make a tiny bit of a mess. Not be a complete pig but still if one is alone, one doesn’t have to pay attention, if one is in public one has to be a little more delicate in ones eating activities. Yeah I think I’m a little better mannered when I’m out in public than I am by myself yeah.”*

Participants with overweight or obesity also noted overt and perceived expectations to undereat because of their high BMI. As an example of an overt social pressure to undereat, participant L5, who lost a significant amount of weight earlier in life, said the following about stigmatization from their family and friends: *“A lot of it was comments, so it’s just like crazy because I would never say those things to people. You know sometimes there’s reasons that they’re struggling with their weight or whatever it is and it’s not just like so black and white. Like, oh you’re being lazy. There’s other reasons that could be happening. And so yeah there was comments but there was also like ya know implications, ya know people implying things. Um yeah people can just be a little too blunt, sometimes.”* Similarly, a focus group participant with obesity described the overt expectations experienced from family as follows: *“Well both her mother and my father’s mother were obese. She I think is terrified that I’m going to be obese and so ever since I was eleven years old she’s hounded me about weight and, you know, are you sure you’re going to eat all of that? And I could go on. …I see them maybe twice a year because of distance, and I enjoy being with them but I always worry, you know, I’m afraid of like … is she watching everything I eat? And silently judging me or later going to tell me…yeah.”*

Regarding perceived expectations to undereat, L5 who is quoted above, talked about their experience: *“I wasn’t happy with my weight. I wasn’t happy with ya know, people do treat you differently if you know they see something that is a problem, that’s like, oh you need to fix that. And like for me it was my weight and I was just self-conscious and I was just tired of family and friends like making comments, ya know implying things. So it was just like, ok I’m sick of this I need to do this and I need to feel good about myself.”* However, these perceived expectations were not necessarily shared by participants who currently have overweight or obesity. Instead, the perceived social expectation that was expressed more frequently was eating behavior modification in social settings, as expressed by participant OB7: *“I think one naturally kind of pays attention to sort of how energetic, how ravenous somebody is. So I may try to you know be a little more polite when I’m with others than I might be with myself. And if, I will notice someone who doesn’t enjoy food as much as I do.”*

## 4. Discussion

**Figure 2:**
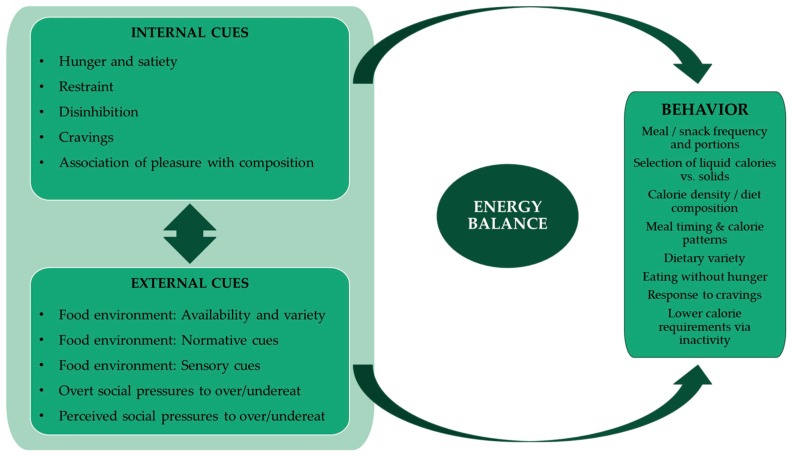
Model for interplay of external and internal factors modulating overeating and undereating.

Individuals with overweight or obesity have been hypothesized to be particularly susceptible to environmental cues that promote overeating [18,19,35,36,37,38]. Based on previous work, these cues include large portions [4,12,13,14] and high dietary variety [9,10,11]. However, the emphasis on ‘cues’ within the physical environment implies participant-driven responsiveness to the environment, whereas the present study indicates that other types of external factors are also important, including those arising from overt social pressures and perceived social expectations. Characterization of these additional external factors highlights the complexity of social stigmatization and perhaps cognitive dissonance regarding the effect of unhealthy eating behaviors on weight management. Based on the data collected in this qualitative study, we defined five sub-groups of external factors experienced by participants, which may contribute to overeating and challenges in weight management, and that should be investigated further in future studies. These included the known environmental cues (e.g., food availability and variety), aberrant normative values implied by excessive portion sizes, and individual perceptions of the palatability of different foods. In addition, overt social pressures and perceived social expectations to overeat in social situations were strongly identified as factors that challenge healthy eating patterns by most participants, but particularly by participants with overweight or obesity, who also indicated they had no effective counter-responses. A summary of internal and external factors identified by our participants and their relationship to known proximal food-related factors determining energy intake is summarized in Figure 2. By explicitly defining five sub-groups of external factors that challenge healthy weight regulation, as indicated in the figure, this study identifies novel targets to address in interventions for healthy weight management. 

Work from our laboratory and others has demonstrated that a high level of disinhibition (defined as overeating in response to environmental and other cues) is associated with obesity and high weight gain over 20–30 years [39,40,41]. As noted by others [42] traditional internal factors (e.g., disinhibition) and external cues (such as food availability) influence each other, and their relative effect on behavior is therefore hard to separate. However, the association between changes in the food environment and the obesity epidemic is undeniable [35], and the present work validates their existence and influence. Furthermore, our work is consistent with the suggestion that the influence of external factors on overeating may reflect not only a greater susceptibility of individuals with overweight and obesity to different types of challenges, but also that individuals with overweight or obesity may be subject to environmental factors of *greater magnitude*. This may be a consequence of clustering of overweight and obesity in social networks [43] within which individuals with overweight or obesity have been shown to have increased energy intake on average [44], associated with increased exposure to dietary energy, higher normative portion sizes, and perhaps more overt pressures and perceived expectations to match the dietary energy consumption of others.

Stigma-related stress was a recurring theme in the responses of participants with overweight and obesity, as seen by their concern about being judged when eating in public, comfort when eating alone, perceived and overt expectations from friends/family to overeat or manage their weight, and the experienced tensions between hedonic and healthy eating. This stigma-related stress was also mentioned by a participant who is formerly overweight and recently lost a significant amount of weight. These are pervasive issues that have also been identified by others [45,46], and should be considered in the development of weight management strategies.

This study also confirmed the importance of hunger, lack of satiety, and food cravings as factors contributing to overeating in individuals with overweight and obesity. Individuals with a healthy BMI talked about the unpleasantness of feeling “stuffed” and enjoying food like pizza but generally eating for health rather than indulgence, or viewing health and pleasure as equivalent. Conversely, participants with overweight or obesity generally noted frequently feeling hungry, preferring unhealthy food while in some cases actively disliking healthier options, and seeing these as fundamental challenges to weight management. Such differences may have innate, body, and food- and body-fat-mediate origins. The ice-breaker exercise given to participants in the informant interview component of this study showed that individuals with overweight or obesity tend to prefer foods that are higher in energy density, and such foods are known to be less satiating when consumed in energy-controlled portions [47,48,49]. Furthermore, the energy content of preferred foods by the overweight obese/group may be underestimated, as the research setting (a nutrition research center) may have skewed some of the participant responses toward healthier choices. In fact, some participants stated preferring foods with higher caloric content but made healthier choices as part of their final selection. Nevertheless, the possibility of social desirability bias in the participants with healthy BMI cannot be excluded. Future studies with larger sample size and neutral settings should investigate this question of food preference. 

While there is clearly a genetic component to body fatness [50], additional factors may contribute to excess body fat, as proposed by the carbohydrate-insulin model of obesity, whereby higher dietary glycemic load leads to a higher insulin-to-glucagon ratio and shifts the metabolic balance toward energy storage [51]. A preference for high-calorie foods via leptin resistance in the reward centers of the brain [52] may be an additional contributing factor, that can be reversed with weight loss [53]. 

The strengths of this study included development and administration of the questions by a team of nutrition experts who had sufficient background knowledge to support follow-up on participant responses in both interviews and focus groups, the use of both focus groups and informant interviews for the participants with overweight or obesity, and the wide range of BMI levels in both men and women. Weaknesses include the fact that a small convenience sample of participants mainly with overweight and obesity was obtained. Further studies are needed to determine the generalizability of the results and the replicability of the comparisons summarized here according to weight status. Given the numbers of participants by BMI category and the inability to differentiate responses by BMI category in the focus groups, participants with overweight and obesity had to be considered as a single group, and future studies should compare different BMI categories. Furthermore, this study did not account for body composition or healthy overweight, which is a limitation to be addressed in future research. As mentioned above, it is possible that the study setting may have introduced bias in participant responses. It is also important to note that the attitudes and behaviors to internal and external cues identified here were not entirely uniform according to BMI group, suggesting that studies with increased subject number and additional individual information (such as a more thorough description of weight history, cultural background, and biological status) should be considered in order to better understand the underlying factors of overweight and develop effective strategies for its treatment.

## 5. Conclusions

This study delineated five domains of external factors influencing food intake. These included not only the recognized environmental cues, food palatability, and normative values for portion size, but also overt social pressures to overeat, and perceived social expectations to overeat. Future studies should include larger sample sizes and differentiation between overweight and obesity. The new framework described here can now be further tested and applied to the development of weight management interventions that support participants in dealing with external cues that may lead to eating behaviors associated with overweight and obesity.

## Figures and Tables

**Table 1:** Population characteristics.

Group	Age Mean ± SD	Gender N Female (Male)	BMI Mean ± SD
Interviews: Healthy BMI (L, *n* = 5)	59 ± 17	4 (1)	21.6 ± 2.5
Interviews: Overweight/Obese BMI (OW/OB, *n* = 5)	59 ± 13	2 (3)	32.2 ± 4.0
Focus group 1 Overweight BMI (FG1OW, *n* = 7)	61 ± 18	5 (2)	26.0 ± 1.2
Focus group 2 Overweight/Obese BMI (FG2OWOB, *n* = 3)	42 ± 7	1 (2)	30.5 ± 2.3
Focus group 3: Overweight/Obese BMI (FG3OWOB, *n* = 4)	63 ± 13	4 (0)	29.3 ± 2.8

**Table 2:** Perceptions and responses to hunger, satiety, and food cravings.

Factor	Description	Healthy BMI	Overweight/Obese BMI
Hunger and satiety	Awareness and actions when hunger is experienced	L1: “So I don’t ever really get that hungry cause I’m always kind of, like I’ll take food with me. So I try to avoid getting really hungry because then I know I’ll probably, I might overeat.” L2: “I don’t eat ‘til I’m stuffed so if there is too much food I will, you know, cut it down. I don’t like that feeling of like basically you have to undo your pants.”	OB7: “I don’t have much patience and I do feel hungry frequently and that is probably a problem. I even, yeah I sometimes think of hunger as being the root of my inability to lose weight and I occasionally glance at the ads for products that are being developed that allege that they address hunger. If I could safely take something or even eat in a certain way that would just make me less hungry during the course of the day I would jump at it…I see hunger as being kind of at the root of the problem, the inability to lose weight.“ FG1OW: “I try and drink water but it never works.”
Restraint	Behaviors to prevent overeating	L1: “I don’t buy them all the time, it is a favorite but I just don’t buy it all the time.” L5 (formerly overweight): “I try to eat healthy, so and I have been for a while but, ya know so I try not even to see these foods that like, that taste good um, cause it’s tempting but, um my taste buds gravitate towards croissants and beer and strawberries.”	OB7: "I try to keep really tempting stuff out of my house and out of my reach.” OB9: "I would’ve circled the donut but I’m trying to lose my belly so I’m refraining from eating donuts.”
Disinhibition	Behaviors to enable overeating	L2: “I can indulge a little bit more on the weekends than I do during the week.” L5 (formerly overweight): “I maybe only drink beer maybe once or twice a month. Um, a croissant maybe I’ll have a couple times a month. Um, so just like on a rare occasion. Oh but strawberries I eat that on a regular basis.”	OW6: “At times I certainly eat too much… you make whatever amount … there is and you just eat it. I think that is very common. And maybe I have an idea in my mind what that should be. So it’s maybe more of my brain than in my stomach.” OB10: “I guess I normally those foods are not available to me so I probably see somebody eating um, like some barbeque ribs so I say to me “I try that” or um some potato chips. “Oh I’ll try some of those” or potato salad, you know, I’ll get a scoop of that. But yeah, I guess I’m influenced by what other people eat.”
Cravings	Awareness and actions relating to cravings	L2: “The thing that I really enjoy the most and I don’t buy it because I will eat the whole bag are Cheese Curls. That is my indulgence. I love Cheese Curls!… If I buy them I will only buy a small package because as I said I will eat the whole thing.” L3: “I’ve also allowed myself to indulge more in these healthy, you know, sometimes raspberries can be expensive, I’m not buying any ice cream so um….so I permit myself to indulge in the things that I like.”	OB10: “I’ll get up and I’ll feel hungry, well not necessarily hungry but I feel like eating something and most of the time it’s not necessarily hungry.” OB8: “Uhhh. Yea, if it’s something I really enjoy, I don’t have to be hungry to eat it. I would just eat it. Not out of need to eat it … like my cottage cheese… So I might go in the fridgerator and grab the tub of cottage cheese and I might sit down there and eat it.”
Association between pleasure and food composition	Tension between food perceived as healthy, and food palatability	L3: “I’d say [health] that’s probably primary, that is, within the range of healthy food, then taste, and um for both of us but that is keeping within the range of healthy food… I rarely cook food that I don’t like.” L5 (formerly overweight): “Yeah definitely, I mean if it doesn’t taste good it’s like, I’m really less likely to eat it. So the taste is very important but sometimes I will choose the healthier thing even though, um something else like I know it would taste better, you know what I mean. So that’s kind of tough, um it’s been tough for me to do that.”	FG1OW: “They’re [selected foods] high sugar, high carbs, all the good stuff.” FG1OW: “Ohhh.You can add that to the unhealthy group. That tastes good.” OW6: “Well there are things I enjoy like pizza I am not eating because I don’t think it is as healthy as I would like it to be.“

Representative quotes have been selected from participants with a healthy body mass index (BMI) (interviews), and participants with an overweight or obese BMI (interviews and focus groups).

**Table 3:** Categories of external cues that influence eating behavior.

External Cues	Description		Healthy BMI	Overweight/Obese BMI
Food environment: Availability and variety	Modifying eating behavior according to available amounts and variety of food		L2: “I mean even when I go out to eat I, you know, try to stay away from like the starchy foods. I try to, you know, have instead of a potato I usually have a double serving of vegetables.” L5 (formerly overweight): “And there’s so many choices so that’s also something that you have to deal with.”	OW6: “I’ll eat it now if it’s available. You know, if I walked through some reception party, yes, always.” FG1OW: At celebrations–“And everybody makes something different. Something that we usually don’t have.”
Food environment: Normative cues	Response to environmental indicators of eating (e.g., portion size, dietary guidelines)		L5 (formerly overweight): “I won’t want a portion that’s too big, it’s like if you’re at a restaurant they give you so much, it’s like I really don’t need that much, maybe I could like bring leftovers, I’ll bring some of it home or whatever. So it‘s portion definitely.” L3: “I’m pretty aware of what stuff you ought to eat and what stuff you ought to avoid and I try to make our meals healthy.“	OB10: “Um eating well, eating healthy and also um, um, portion size is a big thing to me and I struggle with it…Well if they say this is a single serving and I believe it, I’m like “that can’t be a single serving” so I tend to eat more than recommendations, you know, so having said this is a single serving, I’ll probably eat three times that amount… Right, it’s too little. And so I struggle with portion sizes. So I tend, that’s why I try to eat things that are health in my mind so I can eat a little bit more because its healthy.“ OB8: “Oh, I think nutrition is important because you have to be careful that you’re not eating too much fat. You have to be careful that you’re not eating foods that are high in salt content, high in sugar content. And you gotta eat a balance of food. A balance of green, leafy vegetables, squash and green peppers. You have to eat that balance of food because that’s where your nutrition’s gonna come from, a balance of food.”
Food environment: Sensory cues	Characteristics of food that modify eating behavior		L5: “How healthy it is or unhealthy, um that’s the top priority.“ L2: “It’s the corn, chicken cutlet, it has cheese in it. It looked good so I bought it.”	OB8: “Cause food’s gotta taste good in order to enjoy it. Has to taste good, has to be appealing. I try to go, if I go to restaurants, I’m going to restaurants that are known for the quality of their food.” FG3OWOB: “I think eating things that I find delicious and just enjoyable. I mean I eat, I love eating. I love food. And just… I get a lot of pleasure. You know, eating well to me can be like having a ginormous salad with all kinds of goodies in it or it can mean having a nice hot slice of pizza. It doesn’t necessarily…not so much defined by how nutritious it is, but is it something that makes me feel happy. And sometimes I feel like a salad makes me happy. Sometimes I feel like a donut makes me happy, or what have you. But that it’s satisfying and, you know, kind of like you finish it and you’re like “okay, that was worth it”.”
Overt social pressures to overeat or undereat	Comments and behaviors of family, friends and others that influence overeating or undereating	Overeat	L2: When eating out–“And they watch what I eat too and they’re like “Oh, so you’re not having potatoes, you’re just having vegetables”.” L3: “But at a celebration I feel like I ought to have a drink, again I will go ask for seltzer with lime because everybody assumes you’re drinking a gin and tonic.”	OB9: “It depends on the situation. Like if I’m somewhere and I know I’m gonna be insulting them if I don’t eat it. I’d swallow without chewing. That’s about it and I’d never try again. Like I tried salad before and never again. Like little things like that. Roast beef at a friend’s house and I didn’t want to be insulting and say “oh I don’t eat that.” So I just swallowed it and didn’t even chew it.“ FG2OWOB: “It’s Southern culture, you can’t go somewhere and not eat.”
		Undereat	L3: “My doctor told me “don’t eat things with added sugar”.” L5: “A lot of it was comments, so it’s just like crazy because I would never say those things to people. You know sometimes there’s reasons that they’re struggling with their weight or whatever it is and it’s not just like so black and white. Like, oh you’re being lazy. There’s other reasons that could be happening. And so yeah there was comments but there was also like ya know implications, ya know people implying things. Um yeah people can just be a little too blunt, sometimes.”	OB9: “Well both her mother and my father’s mother were obese. She I think is terrified that I’m going to be obese and so ever since I was eleven years old she’s hounded me about weight and, you know, are you sure you’re going to eat all of that? And I could go on. …I see them maybe twice a year because of distance, and I enjoy being with them but I always worry, you know, I’m afraid of like … is she watching everything I eat? And silently judging me or later going to tell me…yeah.”
Perceived social pressures to overeat or undereat	Feelings about being judged by others that influence overeating or undereating	Overeat	L1: “But I actually don’t like talking and eating. I mean I do it, you do it in certain settings. But I feel like you’re not paying attention to what you’re eating when you do that and you’re just more into the conversation, you’re just kind of putting everything in your mouth.” Same participant: “So I actually prefer not to do that. I actually prefer to eat alone, cause then I’m like really focused on the food and how much I’m eating and I can actually taste it and enjoy it.” L5: “Um, but depends on the setting like, if I’m out to eat with my friends and they’re all eating dessert, then I’m more likely to get something like that. Um, I guess it depends on the setting, if it’s in a restaurant or at home. Um, like when I’m on my own I’ll just basically eat mostly healthy.”	OB7: “I just think when one is alone, you know you can sort of dive in be you know, be ravenous not you know perhaps make a tiny bit of a mess. Not be a complete pig but still if one is alone, one doesn’t have to pay attention, if one is in public one has to be a little more delicate in one‘s eating activities. Yeah I think I’m a little better mannered when I’m out in public than I am by myself yeah.” OB9: “Yes, nobody’s there to judge you. Nobody’s there to force you to eat something you don’t want to eat. It’s comfortable eating.”
		Undereat	“L3: And I really didn’t need to lose weight but you know, you have a coffee break and everybody is just talking about the Atkins diet. You go out to lunch and everybody is talking about the Atkins diet. It’s like, “okay, I think I’m on the Atkins diet”. I lasted one day. One day haha. I thought “this is just bizarre”.” L5 (formerly overweight): “I wasn’t happy with my weight. I wasn’t happy with ya know, people do treat you differently if you know they see something that is a problem, that’s like, oh you need to fix that. And like for me it was my weight and I was just self-conscious and I was just tired of family and friends like making comments, ya know implying things. So it was just like, ok I’m sick of this I need to do this and I need to feel good about myself.”	OB7: “I think one naturally kind of pays attention to sort of how energetic, how ravenous somebody is. So I may try to you know be a little more polite when I’m with others than I might be with myself. And if, I will notice someone who doesn’t enjoy food as much as I do.” OW6: “And the comments actually we made this big joke…because she kept saying “you look fabulous” and I said, you know, there are three stages of life.I read this: youth, middle age, and you look fabulous. But no one is saying like “you’ve lost weight”. I don’t think hardly anyone is saying that but they keep saying “how fabulous!” and its why. That’s what they are talking about. And it’s just kind of funny.”

Representative quotes have been selected from participants with a healthy BMI (interviews), and participants with an overweight or obese BMI (interviews and focus groups).

## Supplementary Materials

The following are available online at https://www.mdpi.com/2072-6643/11/6/1365/s1, Table S1: Preplanned questions for informant interviews and focus groups; Table S2. Individual age range and BMI category.
