# Supplementary material for: Different and Unequal: A Qualitative Evaluation of Salient Factors Influencing Energy Intake in Adults with Overweight and Obesity
Source: Nutrients. 2019 Jun 18;11(6):1365. doi: 10.3390/nu11061365 (PMC6627170; doi:10.3390/nu11061365)
Supplement: Supplementary file 1 [file nutrients-11-01365-s001.pdf]

nutrients

Different and unequal: a qualitative evaluation of salient factors influencing energy intake in adults with overweight and obesity

Dao *et al*

Supplementary Tables.

Table 1. Preplanned questions for informant interviews and focus groups.

Domain	Question
**1: Ice Breaker**	For informant interviews only: choose your favorite 3 foods from this picture (**Figure 1**). When do you like to eat these foods?
**2: Reasons for eating**	Some people say that what you eat determines who you are; others just use food as sustenance. How does food fit into your life?
**3: Attitudes toward body image, nutrition, health**	What determines your health? What do you have to do to be in good health? In your opinion, does food influence health? How? What are your sources for health or nutrition advice?<br>Do you pay attention to your weight? In your opinion, what are the most effective strategies to lose and maintain weight?<br>If you were not satisfied with your current weight, why would you want to change your weight? Would it be easy to change your body weight if you wanted to?<br>Have you been on weight loss diets in the past? How often and for how long? How did it go?<br>Would anyone make remarks if your weight changed? If yes, who would?
**4: Insularity, fatalism and other general cultural traits**	Nutritionists sometimes talk about "food culture" (how people make choices about what to eat based on their history, identity, people they eat with). What does this idea of "food culture" mean to you?<br>Now we're going to talk about food culture when you were a child. Do you think about food culture the same way you did when you were younger? What are some examples? Give some examples about what you ate as a child, and what has changed?<br>How do you feel about new foods, cuisines and ways to prepare food that you have not been familiar with? On the other hand, how do you feel about foods produced locally where you live or where you are from?
**5: Meal patterns**	What is your definition of a meal? What does not qualify as a meal?<br>On a typical day, how many meals and snacks do you eat per day?
**6: Attitudes to hunger and restraint**	When you feel hungry and it is not time to eat, what do you do?<br>When food is available even if it is not time to eat, what do you do?<br>How full do you usually feel after you eat? Why do usually stop eating?
**7: External pressures to overeat / not eat**	When you attend important life events or celebrations (for example, birthdays, weddings, national holiday celebrations), is there food provided? How do you usually eat at these events? How much food is provided? Do you eat? How much do you eat? Why do you eat?<br>Do you eat differently when you are alone than when you eat with others?<br>Do you feel comfortable eating alone in a public place? Additional probe: How do you feel about eating in front of the TV at home? How do you feel?<br>When you eat with others, are there things / behaviors that you pay attention to? Do you compare your behaviors with others? Can you give some examples?

Table 2. Individual age range and BMI category.

	Participant	Age (y)	BMI category
Interviews: Healthy BMI (N=5)	L1	18-45	Healthy
	L2	45-65	Healthy
	L3	>65	Healthy
	L4	>65	Healthy
	L5	18-45	Healthy
Interviews: Overweight / Obese BMI (N=5)	OW6	45-65	Overweight
	OB7	>65	Obese
	OB8	>65	Obese
	OB9	18-45	Obese
	OB10	45-65	Obese
Focus group 1: Overweight BMI (N=7)	FG1OW-1	>65	Overweight
	FG1 OW-2	>65	Overweight
	FG1OW-3	45-65	Overweight
	FG1OW-4	>65	Overweight
	FG1OW-5	>65	Overweight
	FG1OW-6	45-65	Overweight
	FG1OW-7	18-45	Overweight
Focus group 2: Overweight / Obese BMI (N=3)	FG2OWOB-1	45-65	Obese
	FG2OWOB-2	18-45	Overweight
	FG2OWOB-3	18-45	Obese
Focus group 3: Overweight / Obese BMI (N=4)	FG3OWOB-1	18-45	Obese
	FG3OWOB-2	>65	Overweight
	FG3OWOB-3	>65	Overweight
	FG3OWOB-4	45-65	Obese
